# Analysis of Mitochondrial Function in Cell Membranes as Indicator of Tissue Vulnerability to Drugs in Humans

**DOI:** 10.3390/biomedicines10050980

**Published:** 2022-04-23

**Authors:** Ane Elexpe, Laura Sánchez-Sánchez, Tarson Tolentino-Cortez, Egoitz Astigarraga, María Torrecilla, Gabriel Barreda-Gómez

**Affiliations:** 1 Research and Development Department, IMG Pharma Biotech S.L, 48160 Derio, Spain; ane@imgpharma.com (A.E.); laura.sanchez@imgpharma.com (L.S.-S.); tarson.bil@gmail.com (T.T.-C.); egoitz.astigarraga@imgpharma.com (E.A.); 2 Department of Pharmacology, Faculty of Medicine and Nursing, University of the Basque Country UPV/EHU, 48940 Leioa, Spain; maria.torrecilla@ehu.eus; 3 Institute of Molecular Genetics and Biology (IBGM), University of Valladolid-CSIC, 47003 Valladolid, Spain

**Keywords:** microarray, superoxide, antipsychotic, mitochondria

## Abstract

Drug side effects are one of the main reasons for treatment withdrawal during clinical trials. Reactive oxygen species formation is involved in many of the drug side effects, mainly by interacting with the components of the cellular respiration. Thus, the early detection of these effects in the drug discovery process is a key aspect for the optimization of pharmacological research. To this end, the superoxide formation of a series of drugs and compounds with antidepressant, antipsychotic, anticholinergic, narcotic, and analgesic properties was evaluated in isolated bovine heart membranes and on cell membrane microarrays from a collection of human tissues, together with specific inhibitors of the mitochondrial electron transport chain. Fluphenazine and PB28 promoted similar effects to those of rotenone, but with lower potency, indicating a direct action on mitochondrial complex I. Moreover, nefazodone, a drug withdrawn from the market due to its mitochondrial hepatotoxic effects, evoked the highest superoxide formation in human liver cell membranes, suggesting the potential of this technology to anticipate adverse effects in preclinical phases.

## 1. Introduction

Reactive oxygen species (ROS) formation is a physiological process produced by different pathways and controlled by diverse antioxidant mechanisms; however, it can turn into a pathological state due to an imbalance between oxidant and antioxidant compounds [1]. These compounds are a series of radical and nonradical oxygen species formed upon incomplete oxygen reduction [2] whose augmentation can induce lipid peroxidation and multiple alterations in proteins and nuclei acids. They can be produced by several sources, such as NADPH oxidase (NOX) enzyme family [3], dual oxidase (DUOX) [4], monoamine oxidase (MAO) [5], peroxisomes, and mitochondrial electron transport chain (mETC) [6]. The different types of NADPH oxidases are implicated in reactive oxygen species formation in a variety of tissues, such as brain, heart, or liver [7]; specifically, NOX2, NOX3, and NOX4 are expressed throughout the nervous system [7], whereas other isoforms such as NOX5 are found only in the lymph nodes and spleen [8]. Additionally, dual oxidase enzymes (DUOX) produce hydrogen peroxide directly or indirectly [7]. Monoamine oxidase is an enzyme located in the mitochondrial outer membrane, with two different isoforms (MAO-A and MAO-B). One of its catalytic products is hydrogen peroxide [9,10] whose accumulation provokes the damage of many cell types including neural cells [11] and is implicated in many brain pathologies [12], specifically Alzheimer’s [13] and Parkinson’s disease [14], Friedreich Ataxia [15], multiple sclerosis [16], and some psychotic disorders such as bipolar disorder [17] and schizophrenia [18,19]. Among these proteins, the main ones responsible for cytosolic hydrogen peroxide and other ROS are those involved in the mitochondrial electron transport chain and cytochrome P450 enzymatic system [20]. On the one hand, the oxidative phosphorylation (OXPHOS) process, performed in the mitochondrial inner membrane and composed of five enzymatic complexes, is the main source of energy as well as reactive oxygen species. Mitochondria are organelles present in most eukaryotic cells that can perform a variety of metabolic functions [21], such as oxidative phosphorylation [22,23] and metabolite regulation. They are also implicated in homeostatic signaling [22] and lipid biosynthetic pathways [23]. Principally, superoxide is produced by mitochondrial complexes I and III [24]. Their dysregulation leads to an ATP production decrease, an increase in oxidative stress, and may even initiate apoptotic processes leading to drug side effects [25]. On the other hand, cytochrome P450 enzymes are involved in the metabolization of compounds [20]. These enzymes oxygenate organic xenobiotic substrates and catalyze the reduction of molecular oxygen simultaneously; if it is not performed correctly, oxygen uncouples from the substrate and leads to ROS formation [26].

In physiological states, enzymatic and nonenzymatic antioxidant mechanisms regulate the cellular redox status by controlling the production of second messengers [27,28] and transcription factors [29] in diverse signaling pathways. For instance, superoxide dismutase (SOD) scavenges superoxide radicals [29], while catalase [30], glutathione peroxidase, and peroxyredoxin [24] protect the cell from the adverse effects of hydrogen peroxide through its breakdown into water and oxygen. Nevertheless, the exposure to some stress conditions, such as different pathologies or even medication intake, can make these mechanisms insufficient.

In a certain way, many drugs that are being tested in clinical trials are withdrawn in preclinical phases because of their high toxicity due to the formation of reactive oxygen species. Considering that the major source of these small molecules is the mitochondrial electron transport chain, their dysfunction can generate an energetic reduction that can have a particularly pronounced impact on the efficiency of neuronal functions compared to other tissues [31]. Schizophrenia is a cognitive disorder that affects 1% of the population in which dissociation or thought disruption is experienced [32]. This illness denotes alterations in the neurotransmission of dopamine inside the mesolimbic system and mesocortical pathway [33]. Moreover, first-episode schizophrenic patients have decreased SOD activity making them prone to suffer from oxidative stress conditions [34]. It has been observed that some antipsychotics have been associated with mETC inhibition causing an increase in reactive oxygen species formation [35] by complex I inhibition [36]. Complex I inhibition contributes to a further reduction of mitochondrial energy production, which is already limited in some cognitive disorders, such as bipolar disorder [31] and schizophrenia [31,37]. For instance, clozapine can induce oxidative stress and apoptosis in neutrophil cells [38], whereas another antipsychotic drugs, such as pentazocine [39], seem to reduce it. In addition, these oxidative dysbalanced conditions have been related to extrapyramidal adverse effects [40,41].

Oxidative stress is also a contributing factor in other mental disorders, such as depression [42], the most common of this type of disease. It is characterized by apathy, anhedonia, sleep disturbance, and psychomotor retardation [43]. Among the antidepressant drugs, nefazodone, an antagonist of the 5-hydroxitriptamine (5-HT) receptor, was used for several years to treat depression until it was withdrawal from the market. It was reported that nefazodone caused liver toxicity and hepatic failure [44] due to the inhibition of cytochrome P450 [45], interference with OXPHOS enzymes, and generation of reactive oxygen species [44]. Other drugs, such as clozapine or fluphenazine, have also been associated with some hepatic alterations [46] and cardiac alterations such as cardiomyopathy or myocarditis [47].

Alzheimer’s disease, the most common neurodegenerative cause of dementia [48], is another mental illness in which oxidative stress seems to play a particular role [48,49]. This neurogenerative disorder has alterations such as the deposit of amyloid-beta plaques, dysregulation of central nervous system immune response [50], dysfunction of oxidative phosphorylation, and reactive oxygen species generation due to mitochondrial disfunction [51]. Regarding the pathologic effects of Alzheimer’s disease, ligands for sigma 1 and 2 receptors can regulate the CNS immune response and modulate amyloid-beta production [35,50]. Furthermore, it has been reported that sigma receptor ligands strongly increased mitochondrial superoxide radicals [52].

**Figure 1:**
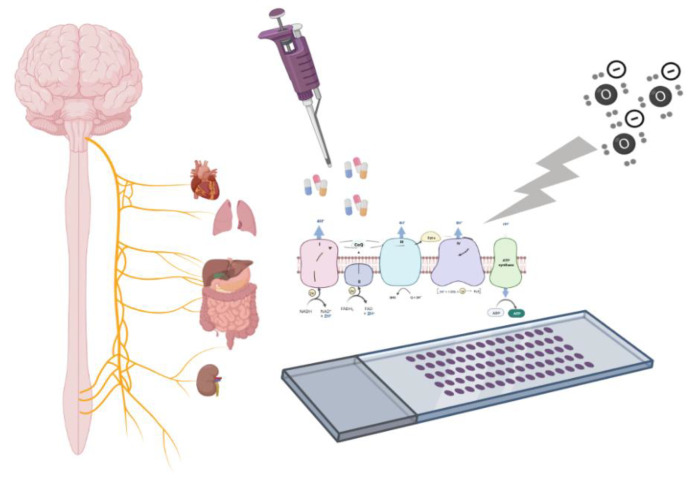
Scheme of the methodology used to evaluate the tissue-specific drug-mediated superoxide formation in human tissues. CMMAs were composed of human cell membranes from 10 different organs and tissues. Drugs and compounds with antipsychotic, antidepressant, anticholinergic, narcotic, and analgesic properties were incubated with CMMAs, and superoxide formation was detected by a colorimetric assay. CMMAs were digitalized, and the data after normalization were analyzed to determine ROS formation in each human tissue.

As these medicines are usually tested in samples from animal models in vitro and in vivo, their side effects could be different to those present in human samples. In this regard, different fluorescence spectroscopy and colorimetric techniques [53] have been carried out to determine the production of ROS in a variety of organisms. In this sense, the aim of this study was to analyze the superoxide formation evoked by drugs and compounds with antipsychotic, anticholinergic, narcotic, and analgesic properties in isolated bovine heart membranes and on human cell membrane microarrays (CMMAs). These CMMAs (Figure 1) consisted of a collection of membranes isolated from 10 human tissues, which maintain the membrane environment and protein functionality, enabling their use in superoxide assays [54].

## 2. Materials and Methods

### 2.1. Drugs and Reagents

Nitrotetrazolium blue chloride (NBT), 3,3′-Diaminobenzidine (DAB), beta-nicotinamide adenine dinucleotide (NADH), sodium succinate dibasic (SDH), decylubiquinone (dUQ), rotenone, antimycin A, sodium azide, cytochrome c from equine heart, olanzapine, and fluphenazine dichloride were purchased from Sigma-Aldrich (Saint Louis, MO, USA). (−)-Pentazocine, PPCC oxalate, PB 28 dihydrochloride, N’N dimethyltryptamine (DMT), BD 1047 dihydrobromide, nefazodone hydrochloride, dextromethorphan hydrochloride, and NE 100 hydrochloride were purchased from Tocris Bioscience (Bristol, UK).

### 2.2. Tissue Samples

Heart samples from *Bos taurus* were supplied by Llodio municipal slaughterhouse (Alava, Spain). Human biopsy tissues were supplied by the AMSBIO (Abingdon, Oxfordshire, UK) tissue bank according to its ethical protocols (Table S2).

### 2.3. Cell Membrane Microarray Fabrication

Cell membrane microarrays were composed of a collection of cell membrane homogenates isolated from different human tissues (liver, jejunum, lung, renal medulla, renal cortex, adrenal gland, myocardium, adipose tissue, duodenum, and spleen). Briefly, samples were homogenized using a disperser (Ultra-Turrax^®^ T10 basic, IKA, Staufen, Germany) or a Teflon-glass grinder (Heidolph RZR 2020, Schwabach, Germany) in 20 volumes of homogenization buffer (1 mM EGTA, 3 mM MgCl_2_, and 50 mM Tris-HCl, pH 7.4) supplemented with 250 mM sucrose. The crude homogenate was subjected to a 1500-rpm centrifugation (AllegraTM X 22R centrifuge, Beckman Coulter, Brea, CA, USA) for 5 min at 4 °C, and the resultant supernatant was collected and centrifuged at 18,000 g (Microfuge^®^ 22R centrifuge, Beckman Coulter, Brea, CA, USA) for 15 min (4 °C). The pellet was washed in 20 volumes of homogenized buffer and recentrifuged under the same conditions. The tubes were finally decanted, and the pellets were frozen at −80 °C, except for one aliquot, which was used to determine the protein concentration. Protein concentration was determined by the Bradford method [55,56] and adjusted to the final concentration.

Membrane homogenates were resuspended in buffer and printed onto glass slides using a noncontact microarrayer (Nanoplotter NP 2.1), placing 3 replicates of each sample (7 nL/spot) onto preactivated glass microscope slides. Membrane homogenates of each tissue were obtained from three different individuals. The printing was carried out under controlled humidity (relative humidity 60%) at a controlled temperature of 4 °C. CMMAs were stored at −20 °C until usage [54]. CMMAs were validated before usage by different methods including Bradford staining for protein determination, enzyme activity assays (NADH oxidoreductase, succinate dehydrogenase, and cytochrome c oxidase), and radioligand binding assays. [54,57,58,59,60,61]

### 2.4. Determination of Drug Effects on Superoxide Formation Promoted by NADH in Isolated Bovine Heart Cell Membranes

The NADH-ubiquinone oxidoreductase activity assay was performed on cell membranes isolated from bovine heart tissue. For this purpose, membrane homogenates (0.1 mg/mL) were incubated in the presence of 0.35 mM NADH and 0.5 mg/mL NBT in phosphate buffer (5 mM; pH 7.4) for 3 h at 25 °C with increasing concentrations (from 0.1 nM to 1 mM) of drugs and compounds with antipsychotic, anticholinergic, narcotic, and analgesic properties (olanzapine, clozapine, desclozapine, fluphenazine, pentazocine, PB 28, DXT, DMT, donepezil, BD 1047, PPCC, and NE 100) in the presence and absence of 50 µM dUQ. The reaction started by the addition of membrane homogenates, and NBT oxidation was measured every 5 min spectrophotometrically at 595 nM in a Multiskan FC microtiter plate reader (Thermo Scientific^®^, Waltham, MA, USA). Biochemical data on NADH oxidoreductase-evoked superoxide production were presented as a percentage of basal activity in the absence of the drug under study, with or without decylubiquinone. Every CMMA had three replicates of each tissue, and all the experiments were performed in duplicate.

### 2.5. Determination of Tissue-Specific Effects of Drugs on Superoxide Formation Triggered by NADH Using Human CMMAs

The NADH-ubiquinone oxidoreductase activity was performed using cell membrane microarrays from a human tissue collection (heart, liver, jejunum, duodenum, renal medulla, renal cortex, adrenal gland, adipose tissue, spleen, and lung). CMMAs were incubated in the presence of 0.35 mM NADH and 0.05 mg/mL NBT in phosphate buffer (5 mM; pH 7.4) for 1 h at 25 °C, with the compounds under study (olanzapine, fluphenazine, pentazocine, PB28, and nefazodone) at 30 µM in the presence or absence of 50 µM dUQ. The reaction was started by the addition of the reagents to the CMMAs. After the incubation time, the reaction was stopped by a dipping in dH_2_O. Once dried, the CMMA color signal was acquired with an Epson V750 pro scanner, and digital images were analyzed with the software Adobe Photoshop CS5 (Adobe Systems Incorporated, Mountain View, CA, USA) and quantified using software ImageScanner (IMG Pharma S.L, Derio, Spain).

### 2.6. Determination of Tissue-Specific Effect on Superoxide Formation Induced by Succinate Using Human CMMAs

Succinate dehydrogenase activity was performed on cell membrane microarrays from a human tissue collection (heart, liver, jejunum, duodenum, renal medulla, renal cortex, adrenal gland, adipose tissue, spleen, and lung). CMMAs were incubated in the presence of 1 mM succinate, 0.05 mg/mL NBT, and 50 µM dUQ in phosphate buffer (5 mM; pH 7.4) for 16 h at 25 °C. The reaction was started by the addition of the reagents to the CMMAs. After the incubation time, the reaction was stopped by a dipping in dH_2_O. Once dried, the CMMA color signal was acquired with an Epson V750 pro scanner, and digital images were analyzed with the software Adobe Photoshop CS5 (Adobe Systems Incorporated, Mountain View, CA, USA) and quantified using software ImageScanner (IMG Pharma S.L, Derio, Spain).

### 2.7. Determination of Drug Tissue-Specific Effect on Cytochrome C Oxidase Activity

Cytochrome c oxidase activity was assayed on cell membrane microarrays from a human tissue collection. CMMAs were incubated in the presence of 1.3 mM of DAB and 0.01% of cytochrome c in phosphate buffer (0.1 M; pH 7.4) for 16 h at 37 °C in darkness. After the incubation time, the reaction was stopped by a dipping in dH_2_O. Once dried, the CMMA color signal was acquired with an Epson V750 pro scanner, and digital images were analyzed with the software Adobe Photoshop CS5 (Adobe Systems Incorporated, Mountain View, CA, USA) and quantified using software ImageScanner (IMG Pharma S.L, Derio, Spain).

### 2.8. Data Analysis and Normalization

Data handling and analysis were carried out using Excel and GraphPad software (version 9.2). The identification of outliers was carried out applying the following formulas:

$$\mathrm{CV}=\frac{\mathrm{SD}}{\bar{X}}$$

$$Y_{1}=\bar{X}-\mathrm{DF}\;\times \mathrm{SD}$$

$$Y_{2}=\bar{X}+\mathrm{DF}\times \mathrm{SD}$$

SD = standard deviation; DF = deviation factor; and CV = variation coefficient.

Points were identified as outliers and excluded if the variation coefficient (CV) was higher than 0.15, Y_1_ was higher than the point analyzed, or Y_2_ was lower than the point examined. We used a deviation factor of 1 in our analysis. In experiments performed with bovine heart membranes homogenates, a nonlinear analysis (log (agonist) vs. response and log (inhibitor) vs. response) was performed.

For microarrays, the analysis data obtained were normalized to the amount of total protein and were expressed as means of independent data points ± S.E.M. The normality of the data was tested using Shapiro–Wilk statistical test with α: 0.05. For Gaussian distributed data, a statistical analysis was performed by one-way, two-tailed ANOVA with Tukey’s multiple comparison test. To analyze nonparametrical data, the Kruskal–Wallis test with Dunn’s multiple comparison test was performed. Statistical differences were indicated by *p*-values ≤ 0.05.

## 3. Results

### 3.1. Protocol Optimization of Superoxide Formation in Bovine Heart Membranes Homogenates

The effect of the different compounds studied relating to superoxide formation was determined in isolated bovine heart membranes following the protocol described in the materials and methods section.

**Figure 2:**
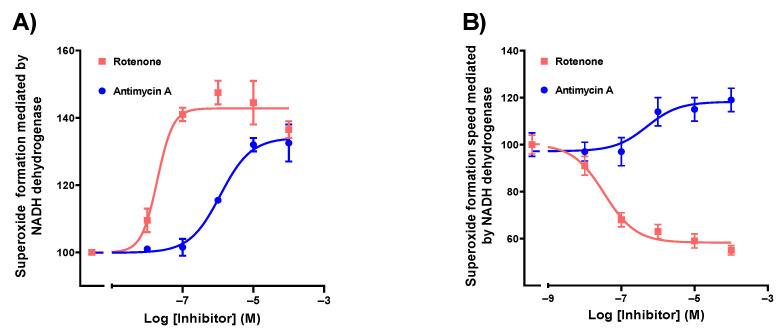
(**A**) Concentration–response curves obtained from the maximum superoxide formation evoked by NADH in the presence of mitochondrial electron transport chain inhibitors of the complex I (rotenone) and III (Antimycin A). (**B**) Superoxide formation rate evoked by NADH in the presence of rotenone and antimycin A determined from the lineal range of the kinetic assays. (**A**) Nonlinear regression was performed with log(agonist) vs. response model and least squares regression as fitting method. (**B**) Nonlinear regression was performed with log(inhibitor) vs. response model for rotenone and log(agonist) vs. response model for antimycin A. In both cases, least squares regression was used as fitting method.

Firstly, succinate dehydrogenase activity assays were performed determining DCIP reduction (Figure S1), and NADH consumption assays (Figure S2) were performed to ensure that membranes were preserved and functional. After validation, the formation of superoxide in these membranes was evaluated by determining the reduction of NBT in the presence of NADH as substrate, with or without decylubiquinone (Figure S3) to study NADH dehydrogenase activity (complex I). As expected, the presence of the decylubiquinone transporter increased the superoxide formation rate. Moreover, mETC superoxide generation was tested in the presence of rotenone (5 μM) and antimycin A (5 μM) as inhibitors of complexes I and III, respectively (Figure S3). Afterwards, NADH dehydrogenase activity assays were performed with different concentrations of these inhibitors. To determine the maximum effect, dose–response curves were plotted, and nonlinear regression was used for each one (Figure 2A, Table 1). Moreover, superoxide formation velocities were determined for each concentration; a dose–response curve was then plotted, and nonlinear regression was used in order to calculate the maximum superoxide formation velocity (Figure 2B, Table 1).

Both inhibitors increased the total amount of superoxide produced (42.8% rotenone vs. 34.2% antimycin A) but with different potency (Figure 2A, Table 1). By contrast, their effects on reaction velocity were substantially different as rotenone reduced it, whereas antimycin A increased it (Figure 2B, Table 1).

Maximum velocities were calculated by a nonlinear model using a velocity vs. log [inhibitor] curve (Figure 2B). The rates at each concentration were achieved from a lineal range of superoxide formation assays (Figure S3) determining the slopes for every different inhibitor concentration. To calculate pharmacological parameters, we used these velocities and the maximum effect (Emax) obtained from the kinetic dose–response curves (Figure 2B).

### 3.2. Effect of Drugs and Compounds on Superoxide Formation in Isolated Bovine Heart Membranes

The capacity of superoxide formation of 12 different medicines with antipsychotic, anticholinergic, narcotic, and analgesic properties was assessed: BD1047, PB28, NE100, and PPCC as sigma receptor antagonists; fluphenazine, pentazocine, olanzapine, clozapine, and desclozapine as first and second-generation antipsychotics; and N, *N*-dimethyltryptamine (DMT), donepezil, and dextromethorphan (DXT) as other drugs with neurological actions.

**Figure 3:**
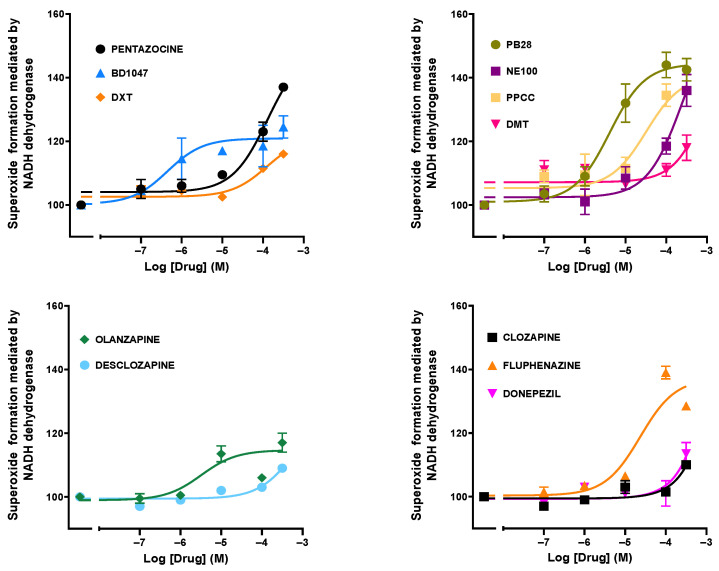
Concentration–response curves of superoxide formation in isolated bovine heart membranes induced by NADH dehydrogenase modulation with pentazocine, BD1047, DXT, PB28, NE100, PPCC, DMT, olanzapine, desclozapine, clozapine, fluphenazine, and donepezil. The superoxide formation promoted by NADH dehydrogenase is represented in percentages versus the activity measured in absence of the tested drug. Nonlinear regression was performed with the log (agonist) vs. response (three parameters) model and least squares regression as fitting method.

An increase in reactive oxygen species formation was observed with certain drugs, namely, NE100 (sigma 1 receptor antagonist), pentazocine (analgesic), and PB28 (sigma 2 agonist) with the higher maximum effect of 60.1%, 45.5%, and 43.4%, respectively (Figure 3; Table 2). On the other hand, PPCC (sigma 1 receptor agonist) and fluphenazine (antipsychotic) reached an Emax of 35.4% and 36.8%, respectively. Finally, the ones with a lower Emax were BD1047 (sigma 1 receptor antagonist), olanzapine (antipsychotic), and DXT (antitussive). DMT (sigma 1 receptor agonist), clozapine (antipsychotic), and desclozapine (antipsychotic) did not promote any effect.

Regarding the rate of reactive oxygen species formation, most of the drugs studied reduced the rate at high concentrations, except for DXT, olanzapine, clozapine, and desclozapine, which did not affect it (Figure S4). The kinetic parameters were determined, and a reduction in the rate of superoxide formation promoted by fluphenazine and PB28 was observed, reaching 51.9% and 34.0%, respectively. Pentazocine, PPCC, and donepezil also promoted a rate reduction of 23.9%, 21.6%, and 17.3%, respectively (Table S1). By contrast, clozapine, desclozapine, olanzapine, DMT, DXT, and BD1047 did not significantly change the rate of superoxide formation evoked by NADH dehydrogenase.

### 3.3. Tissue-Specific Effects of Drugs on Superoxide Formation Using CMMAs of Human Tissues

**Figure 4:**
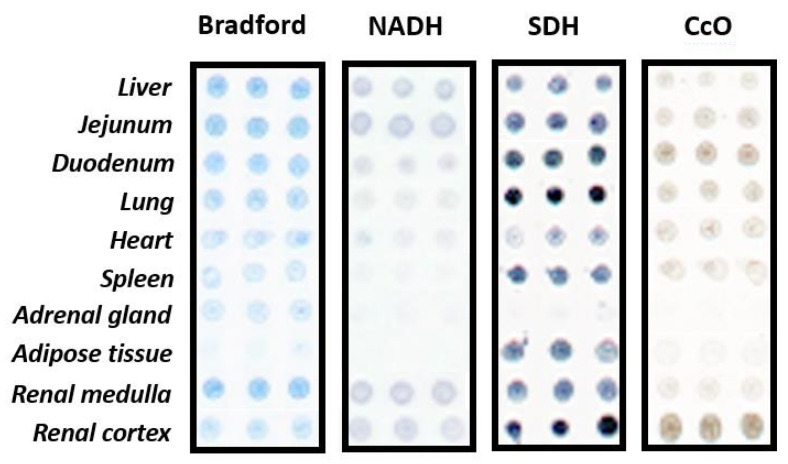
Representative image of human CMMAs showing the immobilized protein (Bradford), the activity of the NADH dehydrogenase (NADH), and succinate dehydrogenase (SDH) together with the activity of citochrome c oxidase (CcO) of the mitochondrial electron transport chain.

To ensure that the immobilized membranes that constitute the CMMAs were functional and the mitochondrial membranes were preserved, the superoxide formation triggered by complex I and complex II substrates and the cytochrome c Oxidase activity were assayed (Figure 4).

**Figure 5:**
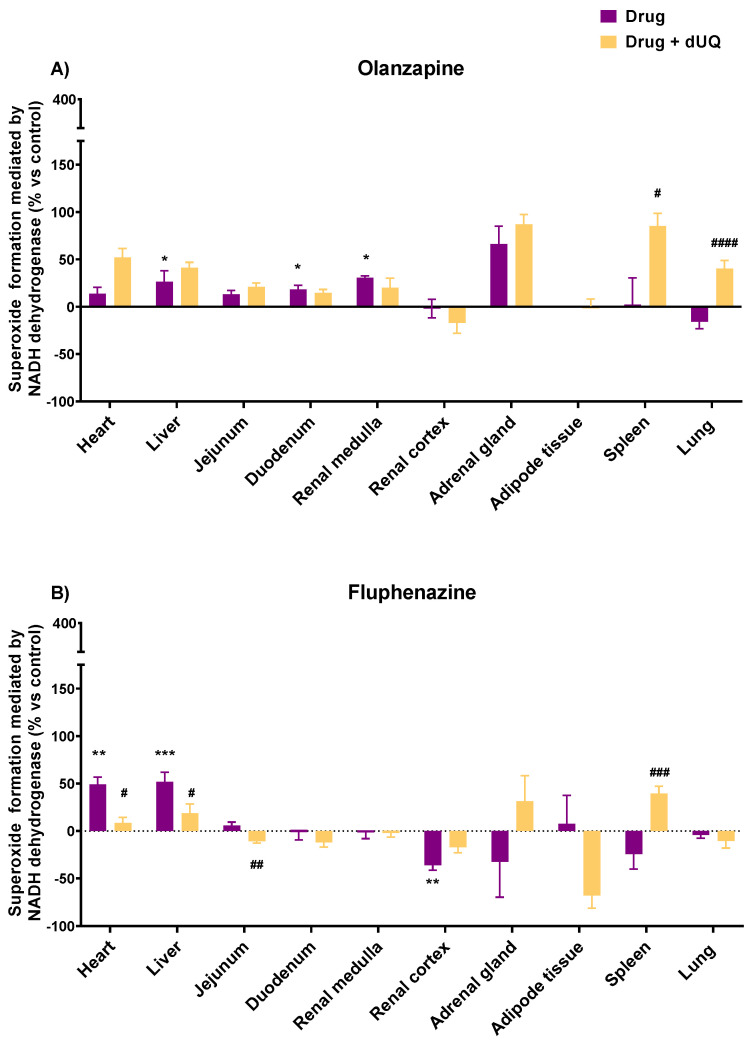
(**A**) NADH-mediated superoxide formation promoted by olanzapine in different human tissues in the presence and absence of decylubiquinone. (**B**) NADH-mediated superoxide formation promoted by fluphenazine in different human tissues in the presence and absence of decylubiquinone. Results expressed as percentages of increase versus the basal activity without the drug (mean ± SEM). Shapiro–Wilk test was performed to test normality. For the tissues that present a Gaussian distribution, one-way ANOVA statistical test with α: 0.05 was performed. For tissues without a normal distribution, Kruskal–Wallis statistical test with α: 0.05 was performed: * *p* < 0.05; ** *p* < 0.01; *** *p* < 0.001 drug vs. control; # *p* < 0.05; ## *p* < 0.01; ### *p* < 0.001; and #### *p* < 0.0001 drug alone vs. drug + dUQ.

**Figure 6:**
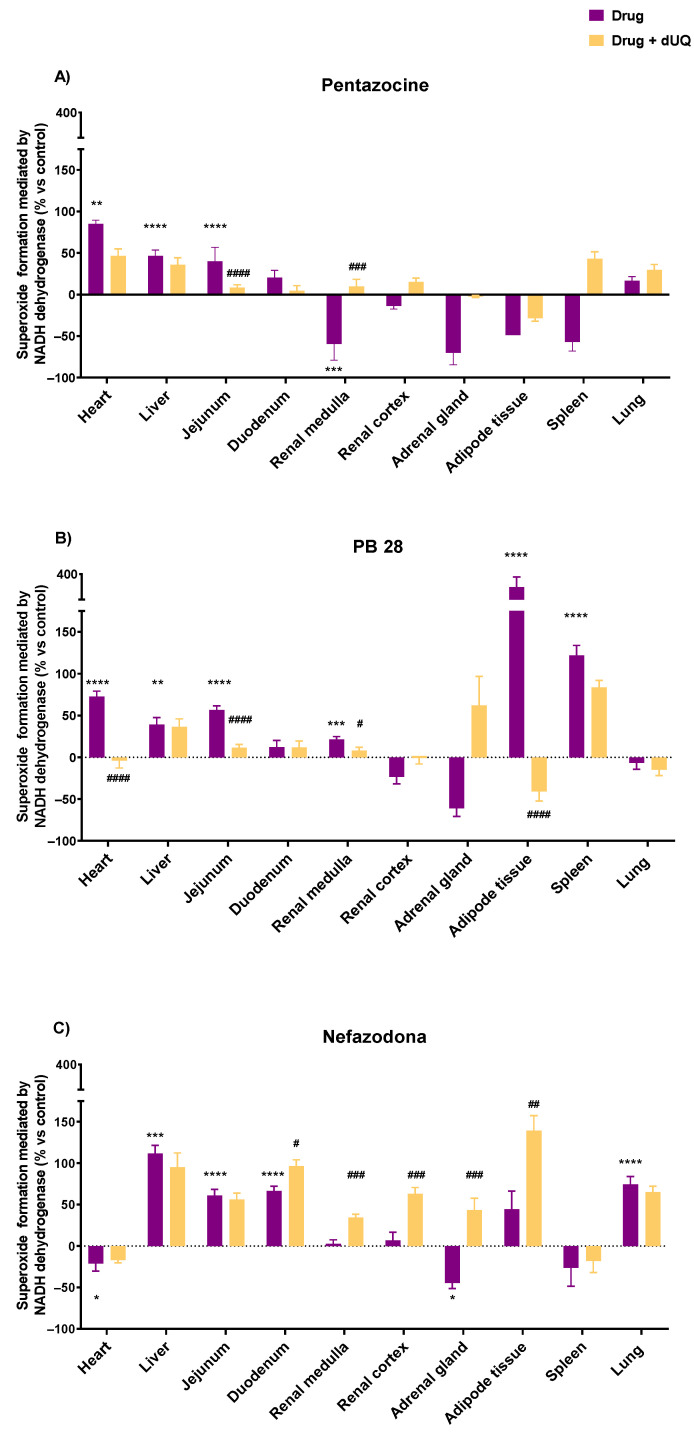
(**A**) NADH-mediated superoxide formation promoted by pentazocine in different human tissues in the presence and absence of decylubiquinone, (**B**) NADH-mediated superoxide formation promoted by PB28 in different human tissues in the presence and absence of decylubiquinone (**C**) NADH-mediated superoxide formation promoted by nefazodone in different human tissues in the presence and absence of decylubiquinone in different human tissues in the presence and absence of decylubiquinone. Results expressed as percentages of increase versus the basal activity without the drug (mean ± SEM). Shapiro–Wilk test was performed to test normality. For the tissues that present a Gaussian distribution, one-way ANOVA statistical test with α: 0.05 was performed. For tissues without a normal distribution, a Kruskal–Wallis statistical test with α: 0.05 was performed. * *p* < 0.05; ** *p* < 0.01; *** *p* < 0.001; **** *p* < 0.0001 drug vs. control; # *p* < 0.05; ## *p* < 0.01; ### *p* < 0.001; and #### *p* < 0.0001 drug alone vs. drug + dUQ.

The selection of drugs tested on human CMMAs was based on the results of the experiments with bovine heart membranes, the information available in the literature, a link between the adverse effects of these drugs, and mitochondrial oxidative stress [62,63]. The drugs selected were fluphenazine, pentazocine, olanzapine, PB28, and nefazodone. In the human heart, all drugs except nefazodone promoted an increase in superoxide formation in the absence of dUQ (13.8% olanzapine <49.1% fluphenazine <72.5% PB28 < 85.0% pentazocine). In the presence of the dUQ electron transporter, olanzapine increased the superoxide formation from 13.8% to 52.0%, while fluphenazine, PB28, and pentazocine reduced it (from 49.1% to 8.3% for fluphenazine; 72.5% to −4.1% for PB28; and 85.0% to 46.5% for pentazocine) (Figure 5 and Figure 6).

Regarding other tissues, olanzapine significantly enhanced the superoxide production in liver, duodenum, adrenal gland, and renal medulla. The presence of dUQ did not alter the effect promoted by olanzapine alone in any of the tissues, except in spleen and lung, where a significant increase in superoxide formation was observed (Figure 5A). Fluphenazine only induced a significant increase in superoxide production in liver and heart, while in all other tissues, it did not enhance and even seemed to decrease it. These actions were blocked by dUQ or even reverted in some tissues, such as spleen (Figure 5B). Pentazocine evoked a substantial increase in superoxide production in heart, liver, and jejunum tissues. A reduction in other tissues, such as renal medulla, adrenal gland, or spleen, was achieved with this compound in the absence of dUQ, while the presence of the transporter reverted these actions (Figure 6A). PB28 increased the superoxide formation in heart, liver, jejunum, renal medulla, adipose tissue, and spleen. However, the coincubation of PB28 with dUQ significantly blocked it in heart, jejunum, renal medulla, and adipose tissue (Figure 6B). Finally, nefazodone caused an increase in superoxide formation in liver, jejunum, duodenum, and lung tissues in the absence of dUQ, while in the presence of this electron transporter, it was not affected, except in the case of the duodenum, where it was also enhanced (Figure 6C). In renal medulla, renal cortex, adrenal gland, and adipose tissue, the coincubation of nefazodone with dUQ evoked an increase in superoxide formation. In contrast, in heart tissue, a reduction of superoxide formation was observed (Figure 6C).

## 4. Discussion

A key aspect of optimizing pharmacological research during the drug discovery process is the early detection of side effects, but the inherent limitations of using human samples delay their study until very late stages. Microarray technology counteracts some of these limitations by producing thousands of microarrays with scarce samples, thus allowing their study in high-throughput screening tests. Cell membrane microarrays have been successfully used to study the lipid fingerprint of nerve and peripheral tissues in animal models and to correlate these data with studies of membrane protein expression and functionality by lipidomic mass spectrometry and immunohistochemical and enzymatic activity assays [59,60]. This technology was used to determine the density and function of membrane receptors after genetic exclusion of the CB1 receptor in animal models with and without transfection of the wild-type CB1 receptor or a mutant (DN22-CB1) in order to shed light on the relationship of cannabinoids, mitochondria, and memory [61]. In addition to G protein-coupled receptors, other membrane proteins remain functional after the immobilization of the membrane homogenates, such as acetylcholinesterase, allowing the study of specific inhibitors. In this sense, tissue-specific activities were assessed in CMMAs consisting of a panel of brain regions to determine the pharmacological profile of anticholinesterase drugs used in the treatment of neurological disorders, such as Alzheimer’s disease [58]. Moreover, protein–protein interactions were studied between programmed cell death ligand 1 (PD-L1) present in membranes from melanoma samples and the programmed cell death protein 1 (PD-1) expressed on T cells, demonstrating the potential of the method to analyze monoclonal antibody drugs and the functional states of immune checkpoint regulators [56]. However, not only is the activity of individual proteins in anchored cell membranes preserved, but so is the interaction among different multiprotein complexes such as those involved in oxidative phosphorylation [64]. In this regard, a tissue-specific activity profile of the mitochondrial electron transport chain was found in CMMAs from different rat brain areas using specific inhibitors of complex I, III, and IV and measuring the NADH-evoked ROS production [54]. The blockage of electron flow through the mETC favors the formation of ROS and oxidative stress and directly reduces cellular energy, which is one of the main causes of adverse effects that can even lead to the withdrawal of drugs from the market.

Thus, in the present study, we describe a novel method based on CMMA to monitor the drug-specific vulnerabilities of certain human tissues to ROS formation using a panel of drugs with antipsychotic, antidepressant, anticholinergic, narcotic, and analgesic properties for validation. For this purpose, the superoxide production was first assessed in isolated bovine heart membranes and finally on cell membrane microarrays from a collection of human tissues. The concentration–response curves obtained in bovine heart membranes for the drugs under study enabled the identification of those drugs that promote an increase in the superoxide formation in this tissue. For their characterization, specific inhibitors of the mETC were used as references. The inhibition of the mETC with rotenone in complex I or with antimycin A in complex III shifts NADH-generated electron transport to reactive oxygen species formation, blocking oxidative phosphorylation [65].

From all the compounds analyzed, pentazocine, olanzapine, fluphenazine, PB28, and nefazodone promoted tissue-specific actions on superoxide formation evoked by NADH dehydrogenase activity in human tissues, the liver and heart being the organs that achieved the highest superoxide formation rates. Both tissues contain a large number of mitochondria, whose dysfunction is crucial in cardiac and hepatic diseases [66,67]. Olanzapine is an atypical antipsychotic drug of first choice in current clinical practice. Unlike pentazocine, this compound did not alter the superoxide formation velocity in bovine heart, although it increased superoxide production by 17%. According to this observation, it has been described that the exposure of human lymphocytes to olanzapine generates an oxidative stress by reactive oxygen species production that is significantly lower than in other drugs, such as buspirone and cetirizine [68]. Moreover, it has also been observed that olanzapine-induced hepatic cytotoxicity [69] is mediated by the overproduction of reactive oxygen species [70], which is consistent with the large increase in superoxide production in human liver membranes determined using CMMAs. However, further studies are needed to identify whether this action of olanzapine is capable by itself of causing oxidative stress in other cell types [71,72,73].

Moreover, an increase in the superoxide formation is reached with pentazocine, a synthetic opioid analgesic especially indicated for the treatment of moderate to severe pain. The increase in superoxide formation evoked by pentazocine in spleen and renal medulla, in the presence of dUQ, may be associated with some allergic reactions and urinary disorders described for this drug [74]. However, pentazocine appears to exert protective actions against oxidative stress and apoptosis in microglia [39,75], although it also modifies NADPH oxidase activity, leading to increased superoxide formation in mitochondria from nonhuman tissues [76] and interacting with other NADH oxidoreductases, such as P450 cytochrome [77]. In this sense, pentazocine highly increased the superoxide production in human heart, which could be related to the stimulatory effect on the cardiovascular system [78] and respiratory depression [79]. Other drugs that modulate sigma opioid receptors (NE100, PB28, PPCC, BD47, and DXT) also triggered superoxide formation in bovine heart membranes with different potency and efficacy by an off-target, nonprimary, G protein-coupled receptor-dependent mechanism. This off-target effect may promote the production of reactive oxygen species that could even trigger cellular stress and cytotoxicity [52]. Fluphenazine and PB28 induced an enhancement of the superoxide formation in bovine heart membranes similar to that produced by rotenone, but with lower potency. Both drugs also increased the superoxide production in human heart and liver tissues but showed significant changes in their effects in other tissues, such as adipose tissue and spleen, where PB28 promoted significant effects compared to the lack of action observed with fluphenazine. It has been described that both drugs exert actions on mitochondrial respiration, although different mechanisms could be involved. In this sense, fluphenazine seems to inhibit brain mitochondrial complex I [80] through the oxidation of essential thiol groups to disulfides, as dithiothreitol, a thiol reductant, restored it [81,82]. In this sense, the increase in superoxide formation evoked by this drug was partially blocked when dUQ was present, suggesting that a competitive mechanism may be taking place at ubiquinone-binding domains. By contrast, PB28 promoted cell death via mitochondrial superoxide production and caspase activation in pancreatic cancer, this effect being partially blocked by the lipid antioxidant α-tocopherol, but not by the hydrophilic N-acetylcysteine (NAC) [52]. Several studies have shown that increased production of reactive oxygen species has a direct impact on homeostasis and cell survival, suggesting that it may be of interest in the treatment of neoplasms [83]. Both compounds reduced the superoxide formation in the presence of dUQ in different tissues such as heart and jejunum. This reduction could be due to a competition for the binding site between the drug and the electron transporter. Thus, the coadministration of these drugs with CoQ during treatment might contribute to the mitigation of side effects derived from these tissues, although a further investigation must be performed.

Every drug tested achieved huge superoxide formation in liver tissues. In this sense, the effect of nefazodone, a serotoninergic-modulating antidepressant [84], had more than double the effect of the other drugs not only alone but also in the presence of dUQ. Nefazodone was withdrawn from the market because it was associated with hepatotoxicity and hepatic failure [85,86] owing to the actions exerted over the cytochrome P450 (CYP3A4) and the complex I of the mETC, which increase the formation of reactive oxygen species [44]. Nefazodone not only had the strongest effect on complex I but also have effect on other complexes [44], decreasing cellular energy levels [79]. In addition to the hepatotoxic effect of nefazodone, which was the reason for its withdrawal, different actions can be observed in other tissues, such as heart tissues. In cell membranes isolated from this tissue, the formation of reactive oxygen species achieved was even lower than in the control, which is in line with the absence of cardiotoxicity previously described in clinical trials [87]. 

In conclusion, the data obtained from the superoxide production assays performed on cell membranes of human tissues provide a safety profile of drugs against oxidative stress, mediated by NADH oxidoreductases, which can be used to anticipate adverse effects that may even lead to their withdrawal from the market. As the stability and functionality of membrane proteins are preserved [54,56,59,61], the potential of this methodology allows complementary studies such as the identification of lipidomic profiles [60] or enzyme activities [58] in multiple tissues and patients to improve the identification of adverse effects at preclinical stages of drug discovery. In our study, this methodology provides relevant data on the oxidative stress triggered by a set of brain-targeting drugs in a collection of human tissues, which are related to certain adverse effects as in the case of nefazodone. Thus, it enables the identification of possible drug side effects in early stages of the drug discovery, before clinical trials get started, optimizing the drug discovery process and improving patient safety.

## Figures and Tables

**Table 1:** Logarithms of half-maximum effective or inhibitory concentrations (*pEC50 or pIC50*), maximum superoxide formation (*Emax*), and maximum superoxide formation velocity (*Vmax*) determined from concentration–response curves for each specific inhibitor.

	Superoxide Formation			
	Maximum Amount		Production Rate	
Rotenone	*pEC50*	−7.7 ± 0.23	*pIC50*	−7.47 ± 0.15
	*Emax*	42.79 ± 4.88	*Vmax*	41.90 ± 2.94
Antimycin A	*pEC50*	−5.94 ± 0.14	*pEC50*	−6.30 ± 0.35
	*Emax*	34.20 ± 2.29	*Vmax*	21.04 ± 3.35

**Table 2:** Potency (pEC50) and maximum effect (Emax) calculated for each drug on superoxide formation evoked by NADH dehydrogenase activity in isolated bovine heart membranes.

	*pEC50*	*Emax* (%)		*pEC50*	*Emax* (%)
PB 28	−5.4 ± 0.2	43.4 ± 3.0	Pentzazocine	−3.9 ±0.3	45.5 ± 11.1
NE 100	−3.6 ± 0.3	60.1 ± 23.1	Donepezil	UD	UD
PPCC	−4.5 ± 0.3	35.4 ± 5.3	Fluphenazine	−4.6 ± 0.3	36.8 ± 4.9
BD 1047	−6.3 ± 0.4	20.6 ± 4.1	Clozapine	UD	UD
DMT	UD	UD	Olanzapine	−5.5 ± 0.4	15.7 ± 3.1
DXT	−3.9 ± 0.3	19.0 ± 5.2	Desclozapine	−3.2 ± 1.0	UD

UD = undetermined.

## Data Availability

The data supporting the findings of this study are available from the corresponding author, Gabriel Barreda-Gómez, upon reasonable request.

## Supplementary Materials

The following supporting information can be downloaded at: https://www.mdpi.com/article/10.3390/biomedicines10050980/s1. Figure S1. DCIP reduction (Abs 595) represented as percentage using succinate (1 mM) as substrate with and without dUQ (50 μM). Assays were performed in bovine heart membranes homogenates. The concentration of 2,6-Dichlorophenolindophenol (DCIP) was 3.2 mM. Figure S2. NADH consumption percentage (Abs 340 nm) using NADH (0.35 mM) as substrate of mETC, decylubiquinone (dUQ) (50 μM), and rotenone (10 μM) and antimycin A (5 μM) as selective mETC inhibitors. Experiment was performed in bovine heart membranes homogenates. Figure S3. Superoxide formation induced by NADH dehydrogenase represented in percentage vs control (basal conditions), with decylubiquinone (50 μM) alone or together with rotenone (10 μM) or antimycin A (5 μM) as selective mETC inhibitors in bovine heart membranes homogenates. Figure S4. Concentration response curves of the NADH dehydrogenase activity on superoxide formation mediated by pentazocine, NE100, PB28, PPCC, BD1047, DMT, DXT, olanzapine, desclozapine, clozapine, fluphenazine and donezepil. The velocity on superoxide formation evoked by NADH dehydrogenaseswas represented in precentajes versus the activity measured in absence of the drug under study. Table S1. Potency (pIC50) and superoxide formation velocity (Vmax) determined for each drug under study evoked by NADH dehydrogenase in isolated bovine heart membranes. Table S2. Information of the different human tissue samples used for CMMAs development.
