# Supplementary material for: Analysis of Mitochondrial Function in Cell Membranes as Indicator of Tissue Vulnerability to Drugs in Humans
Source: Biomedicines. 2022 Apr 23;10(5):980. doi: 10.3390/biomedicines10050980 (PMC9138415; doi:10.3390/biomedicines10050980)
Supplement: Supplementary file 1 [file biomedicines-10-00980-s001.zip › biomedicines-1683833-supplementary.pdf]

biomedicines

MDPI

SUPPORTING INFORMATION

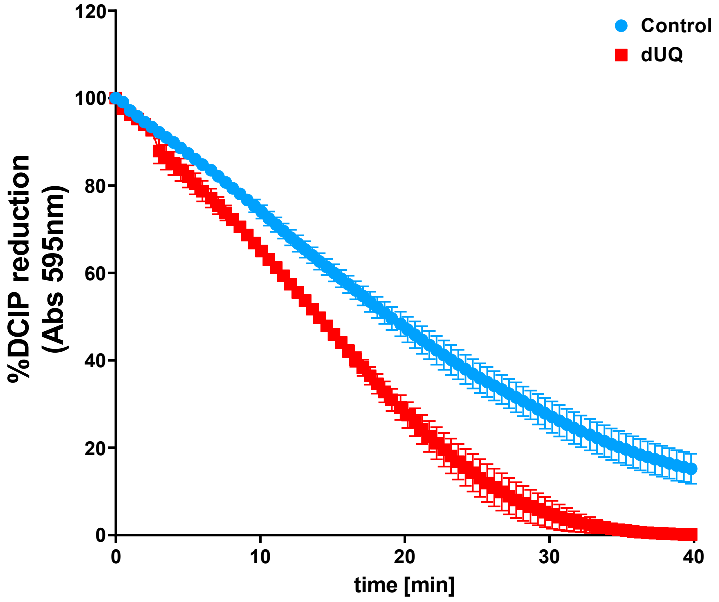

Figure S1. DCIP reduction (Abs 595) represented as percentage using succinate (1 mM) as substrate with and without dUQ (50 µM). Assays were performed in bovine heart membranes homogenates. The concentration of 2,6-Dichlorophenolindophenol (DCIP) was 3.2 mM.

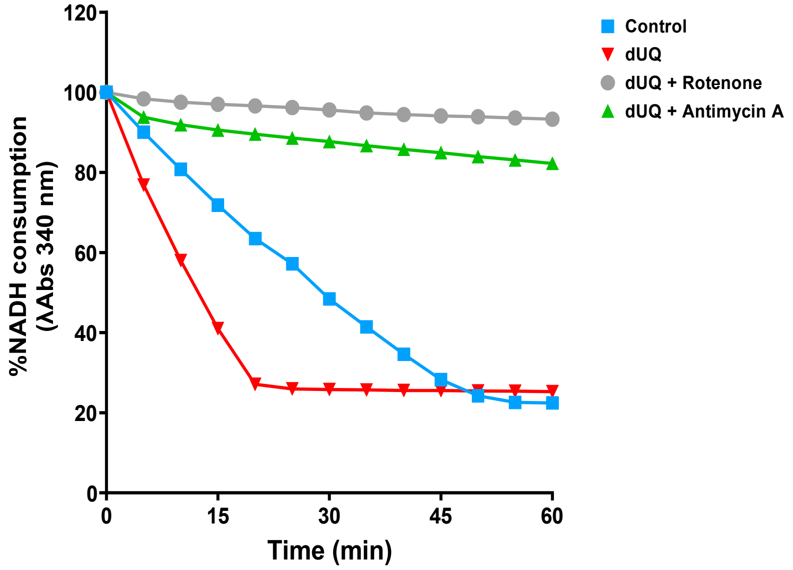

Figure S2. NADH consumption percentage (Abs 340 nm) using NADH (0.35 mM) as substrate of mETC, decylubiquinone (dUQ) (50 µM), and rotenone (10 µM) and antimycin A (5 µM) as selective mETC inhibitors. Experiment was performed in bovine heart membranes homogenates.

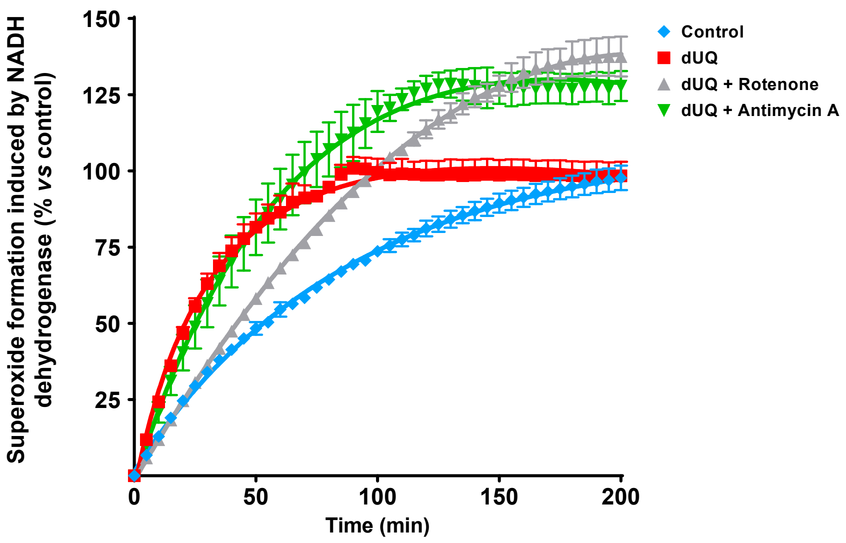

Figure S3. Superoxide formation induced by NADH dehydrogenase represented in percentage *vs* control (basal conditions), with decylubiquinone (50 µM) alone or together with rotenone (10 µM) or antimycin A (5 µM) as selective mETC inhibitors in bovine heart membranes homogenates.

In the experimental conditions used, the inhibition of complex I evoked by rotenone dramatically inhibited the activity of this complex reducing the NADH consumption rate (Figure S2), not only in absence but also in presence of dUQ transporter and decreasing in this way superoxide formation velocity (Figure S3). However, the total amount of superoxide produced per substrate unit, when equilibrium was reached, was higher than in the absence of rotenone, as the main electrons shifted to ROS production rather than continuing through the mETC. By contrast, antimycin A blocks the complex III, so electron flow generated by NADH oxidation can be shifted to reactive oxygen species formation not only in complex III but also in complex I, increasing the rate of superoxide formation. The maximal superoxide production achieved with antimycin A exhibits similar levels to those evoked by rotenone.

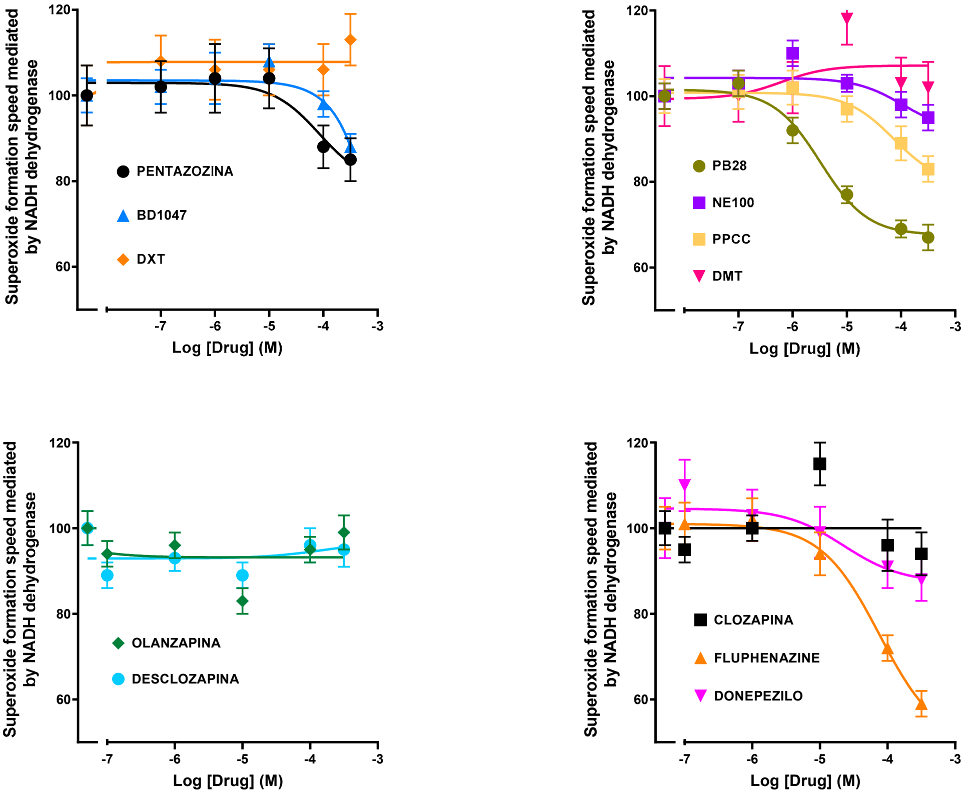

Figure S4. Concentration response curves of the NADH dehydrogenase activity on superoxide formation mediated by pentazocine, NE100, PB28, PPCC, BD1047, DMT, DXT, olanzapine, desclozapine, clozapine, fluphenazine and donezepil. The velocity on superoxide formation evoked by NADH dehydrogenaseswas represented in precentajes *versus* the activity measured in absence of the drug under study.

Table S1. Potency (pIC50) and superoxide formation velocity (Vmax) determined for each drug under study evoked by NADH dehydrogenase in isolated bovine heart membranes.

	pIC50	*Vmax (%)*		*pIC50*	*Vmax (%)*
**PB 28**	-5.5 ± 0.1	34.0 ± 2.0	**Pentazocine**	-4.1 ± 0.5	23.9 ± 7.9
**NE 100**	-4.0 ± 1.3	ND	**Donepezil**	-4.7 ± 0.6	17.3 ± 5.0
**PPCC**	-4.1 ± 0.2	21.6 ± 2.9	**Fluphenazine**	-4.1 ± 0.1	51.9 ± 3.1
**BD 1047**	UD	UD	**Clozapine**	UD	UD
**DMT**	UD	UD	**Olanzapine**	UD	UD
**DXT**	UD	UD	**Desclozapine**	UD	UD

*UD= undetermined

Table S2. Information of the different human tissue samples used for CMMAs development.

Index	Specimen ID	Category	Anatomical Site	Gender	Age	Ethnicity	Cause of death	Procurement Date
1	S13-1 Adrenal Gland	Normal tissues	Adrenal Gland	Male	48	Caucasian (White)	Mechanical Trauma	27/02/2012
2	S7- 1 Adrenal Gland	Normal tissues	Adrenal Gland	Male	47	Caucasian (White)	Acute Coronary Syndrome	11/04/2011
3	S6-1 Adrenal Gland	Normal tissues	Adrenal Gland	Male	46	Caucasian (White)	Mechanical Trauma	18/03/2011
4	S13-18 Duodenum	Normal tissues	Small Intestine, Duodenum	Male	48	Caucasian (White)	Mechanical Trauma	27/02/2012
5	S7-23 Jejenum	Normal tissues	Small Intestine	Male	47	Caucasian (White)	Acute Coronary Syndrome	11/04/2011
6	S6-23 -Jejenum	Normal tissues	Small Intestine	Male	46	Caucasian (White)	Mechanical Trauma	18/03/2011
7	S13-25 Kidney, Cortex	Normal tissues	Kidney, Cortex	Male	48	Caucasian (White)	Mechanical Trauma	27/02/2012
8	90-M-13-28 Kidney, Cortex	Normal tissues	Kidney, Cortex	Male	44	Caucasian (White)	Acute Myocardial Infarction	23/01/2015
9	S8-29 Kidney, Cortex	Normal tissues	Kidney, Cortex	Male	41	Caucasian (White)	Subarachnoid hemorrhage	20/05/2011
10	S13-26 Kidney, Medulla	Normal tissues	Kidney, Medulla	Male	48	Caucasian (White)	Mechanical Trauma	27/02/2012
11	S7-30 Kidney, Medulla	Normal tissues	Kidney, Medulla	Male	47	Caucasian (White)	Acute Coronary Syndrome	11/04/2011
12	S6-30 Kidney, Medulla	Normal tissues	Kidney, Medulla	Male	46	Caucasian (White)	Mechanical Trauma	18/03/2011
13	S6-32 Liver	Normal tissues	Liver	Male	46	Caucasian (White)	Mechanical Trauma	18/03/2011
14	S13-28 Liver	Normal tissues	Liver	Male	48	Caucasian (White)	Mechanical Trauma	27/02/2012
15	S7-32 Liver	Normal tissues	Liver	Male	47	Caucasian (White)	Acute Coronary Syndrome	11/04/2011
16	X-35 Lung	Normal tissues	Lung	Male	56	Caucasian (White)	Traumatic injury	12/02/2009
17	S7-33 Lung	Normal tissues	Lung	Male	47	Caucasian (White)	Acute Coronary Syndrome	11/04/2011
18	S6-33 Lung	Normal tissues	Lung	Male	46	Caucasian (White)	Mechanical Trauma	18/03/2011
19	S13-41 Spleen	Normal tissues	Spleen	Male	48	Caucasian (White)	Mechanical Trauma	27/02/2012
20	S7-47 Spleen	Normal tissues	Spleen	Male	47	Caucasian (White)	Acute Coronary Syndrome	11/04/2011
21	S6-47 Spleen	Normal tissues	Spleen	Male	46	Caucasian (White)	Mechanical Trauma	18/03/2011
22	X-29 Myocardium	Normal tissues	Heart, Myocardium	Male	56	Caucasian (White)	Traumatic injury	12/02/2009
23	90-M-13-26 Myocardium	Normal tissues	Heart, Myocardium	Male	44	Caucasian (White)	Acute Myocardial Infarction	23/01/2015
24	S6-27 Myocardium	Normal tissues	Heart, Myocardium	Male	46	Caucasian (White)	Mechanical Trauma	18/03/2011
25	A-17 Fat Subcutaneous	Normal tissues	Fat, Subcutaneous	Male	50	Caucasian (White)	Ischemic heart disease	14/04/2007
26	X-18 Fat Subcutnaneous	Normal tissues	Fat, Subcutaneous	Male	56	Caucasian (White)	Traumatic injury	01/02/2009
